# Cytological Diagnosis of Pancreatic Solid-Pseudopapillary Neoplasm: A Single-Institution Community Practice Experience

**DOI:** 10.3390/diagnostics12020449

**Published:** 2022-02-09

**Authors:** Brant G. Wang, Haresh Mani, Zoe Q. Wang, Wenping Li

**Affiliations:** Department of Pathology, Inova Fairfax Hospital, 3300 Gallows Road, Falls Church, VA 22042, USA; haresh.mani@inova.org (H.M.); zwang122@alumni.jh.edu (Z.Q.W.); wenping.li@inova.org (W.L.)

**Keywords:** solid-pseudopapillary neoplasm, pancreas, cytology, immunohistochemistry

## Abstract

Introduction. Pancreatic solid-pseudopapillary neoplasm (SPN) is a rare tumor that typically occurs in young females. Although a cytological diagnosis may be easily made in this age group when there are typical features, atypical clinical presentations and unusual cytological features may make this a challenging diagnosis. We present our single-institution experience in a cohort of these tumors, outlining both typical and atypical features. Awareness of unusual clinical and cytological features can help to avoid pitfalls during diagnosis. Methods. We performed a review of all cases of pancreatic SPNs diagnosed over a 15-year period (January 2007 to December 2021). Detailed cytological, clinical, and follow-up histological features were presented and analyzed. Results. Twenty-two cases of SPN were diagnosed at our institution during this 15-year period. Patients ranged from 12 to 73 years of age (mean 33 y, median 26 y) and included 19 females and 3 males. Seventeen patients had cytological material, and fourteen were diagnosed by EUS-FNA. Typical cytological features included papillary clusters with central capillaries, myxoid stroma, monomorphism, cercariform cells, and hyaline globules. Atypical or unusual cytological features that were seen in a few cases were multinucleated giant cells, clear cells, and/or foamy macrophages. A few cases showed features that were similar to pancreatic neuroendocrine tumors (PanNETs). Tumor cells were always positive for β-catenin, CD10, CD56, cyclin-D1, progesterone receptor (PR), and vimentin by immunohistochemistry. They were always negative for chromogranin. Pancytokeratin and synaptophysin stains were positive in 9% and 46% of cases evaluated, respectively. All cases had histological confirmation on resection. The median follow-up duration was 69 months (a range of 2–177 months), with only three cases lost to follow-up. No recurrence or metastasis was identified. Conclusions. We present our experience with cytological diagnoses of SPN in a well-characterized cohort of 22 patients with histological correlation and follow-up data. These tumors occur over a wide range and show varied cytological features. SPNs can be confidently diagnosed on limited cytological material, with limited panel immunohistochemistry aiding diagnosis in atypical cases. Recognizing the associated degenerative changes is crucial in avoiding a misdiagnosis.

## 1. Introduction

Solid-pseudopapillary neoplasm (SPN) of the pancreas is a rare tumor usually seen in young female patients. It is a neoplasm with a low malignant potential, and patients tend to have a good prognosis, especially compared to other malignant pancreatic neoplasms [1,2,3,4,5,6]. A definitive diagnosis is therefore crucial for correct patient management.

The clinical and radiological features of SPNs mimic those of other pancreatic neoplasms. They typically present as solid and cystic masses anywhere along the pancreas [1,2,3,4,5,6]. A preoperative diagnosis can be made on cytology specimens if one keeps this differential in mind. Therefore, cytology is an important tool in the diagnosis and management triage [7,8,9,10,11,12,13,14,15]. We summarize our experience in the cytodiagnosis of SPN, with an emphasis on cytological clues and a simplified panel for confirmatory immunohistochemistry. A few diagnostic pitfalls are also discussed.

## 2. Materials and Methods

We searched files in our Department of Pathology for a diagnosis of SPN over a 15-year period between January 2007 and December 2021. We included both cytology and surgical pathology data in our search. All available material was reviewed. These included Diff-Quik-stained direct smears, Papanicolaou-stained direct smears or ThinPrep prepared slides, hematoxylin and eosin (H&E)-stained cell block sections, and immunohistochemical stains. Emphasis was placed on detailing individual previously defined cytological features including cellularity, pseudopapillary configuration, single cells with a cytoplasmic tail (“cercariform” cells), nuclear grooves/folding, nucleoli, cytoplasmic vacuoles, and intracytoplasmic eosinophilic hyaline globules. Any other findings were also documented. The findings of pertinent immunohistochemistry results were summarized. Follow-up histological resections were reviewed for the correctness of cytological diagnoses, and clinical follow-up information was obtained.

## 3. Results

There were a total of 22 cases of SPN in our files over this 15-year period, including 19 females and 3 males. Patients ranged in age from 12 to 73 years (mean 33, median 26). However, the three males were aged 23 years, 25 years, and 26 years. Patients had presented with abdominal pain or discomfort. One patient (Case #21) had a sports-related trauma, and the tumor was incidentally discovered. The tumors were presented at the pancreatic tail in 12 cases (54.5%), body in 9 cases (41%), and head in 1 case (4.5%). Most tumors (18, 82%) were described as solid and cystic on imaging and gross examination, with three (13.5%) tumors being purely solid, and one (4.5%) being cystic. Tumor sizes ranged from 1.2 to 15 cm. There was no significant association between size and age, gender, or tumor site. Clinical and radiological information, as well as gross examination, is summarized in Table 1.

**Figure 1:**
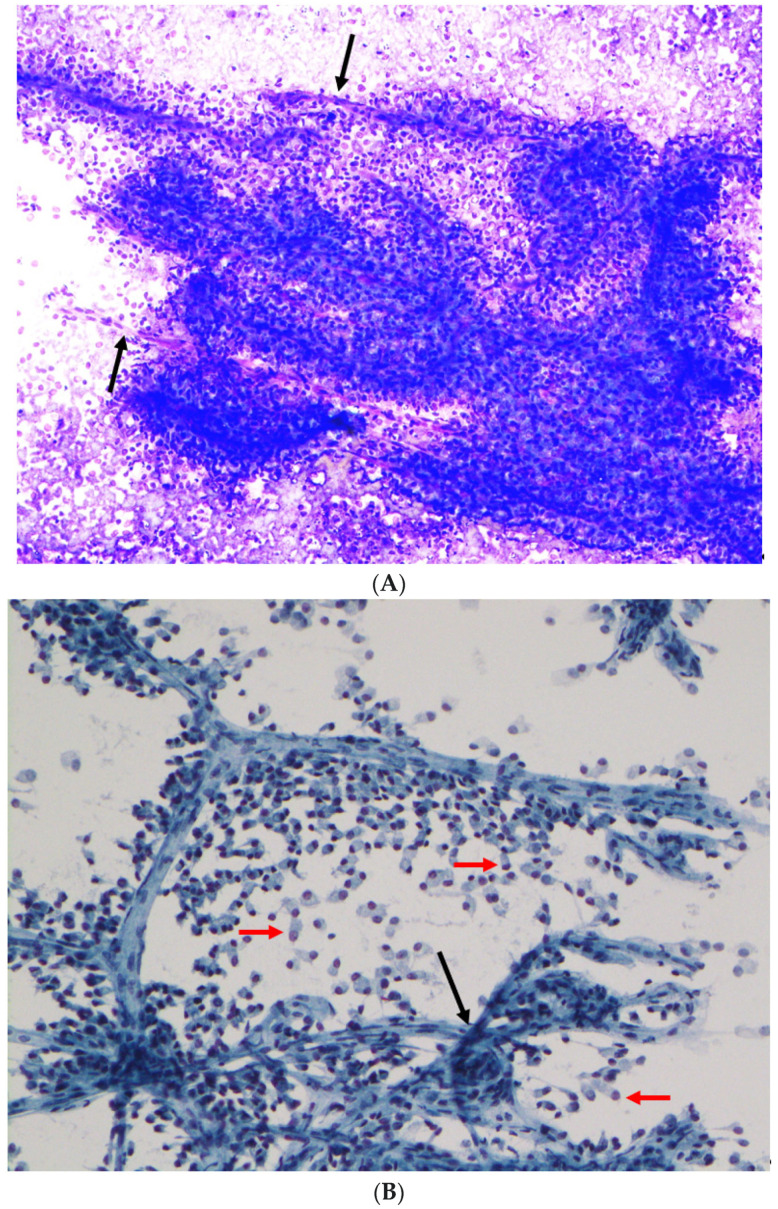

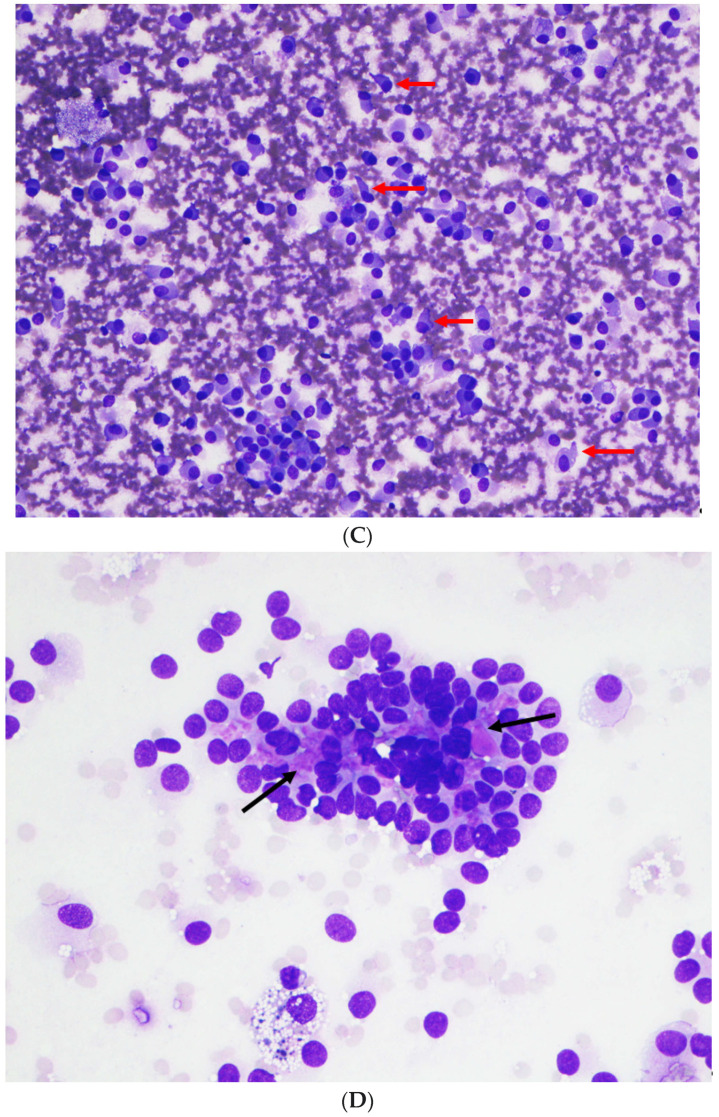

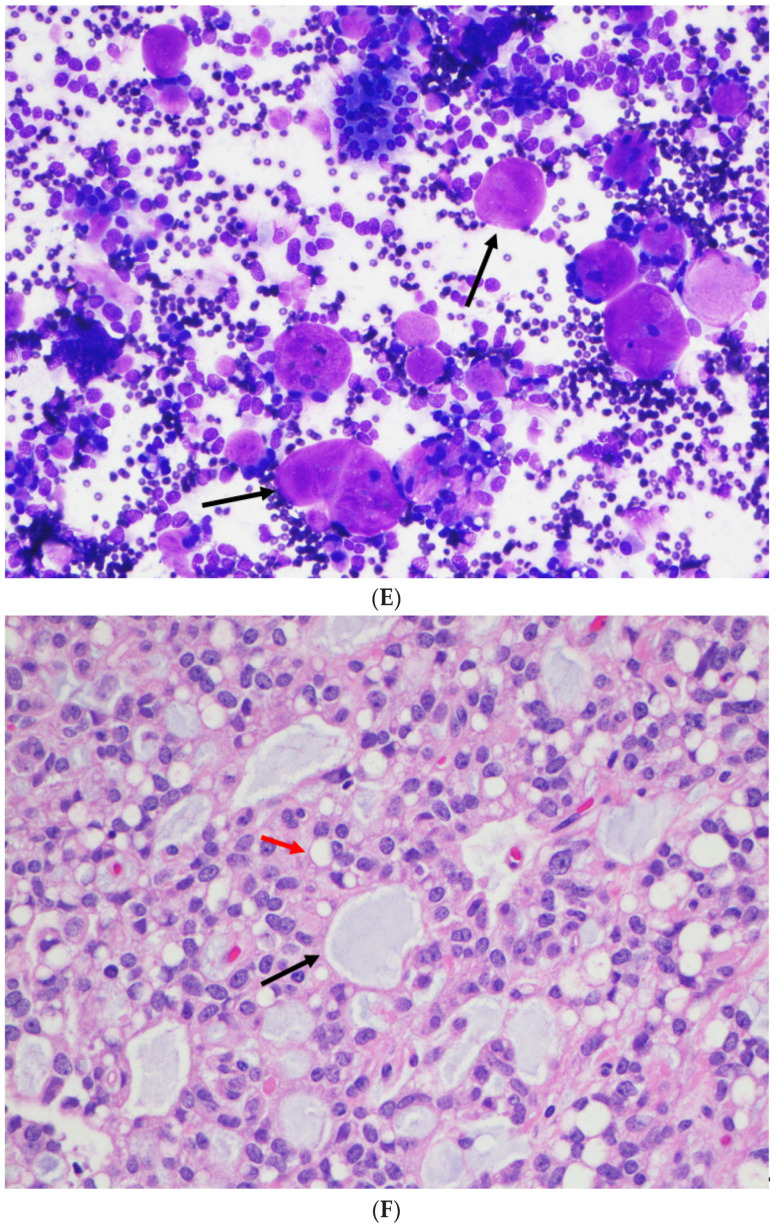

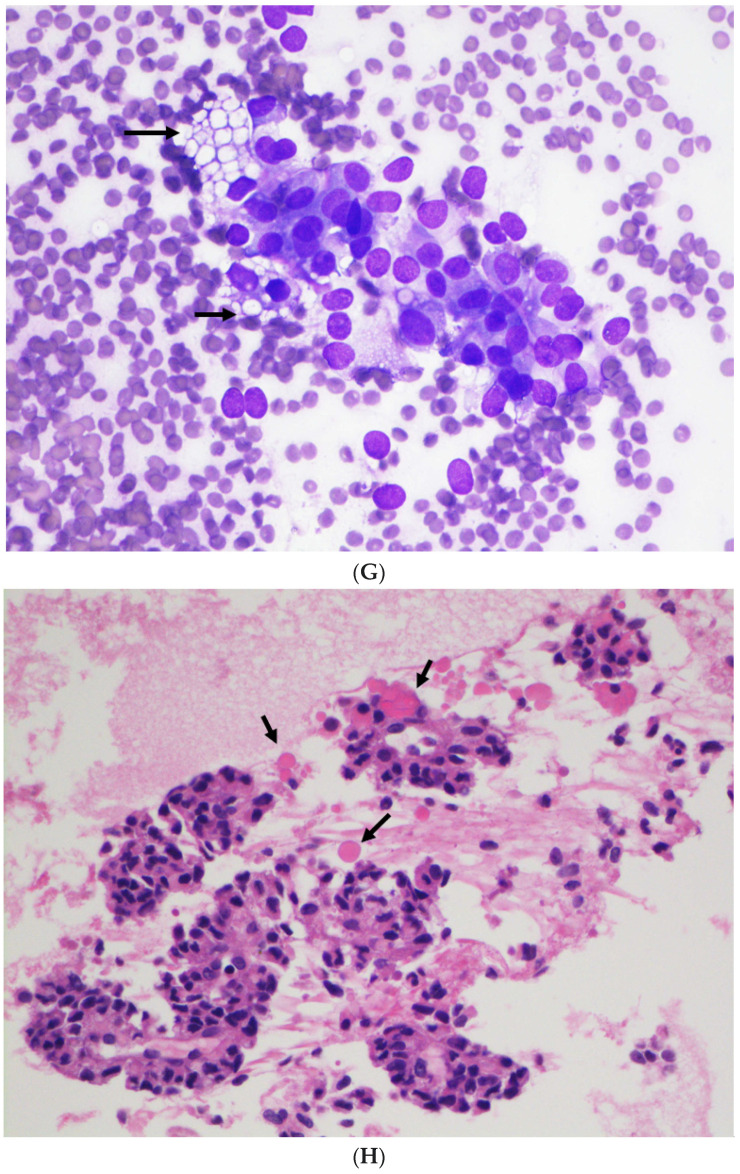
(**A**) Cellular aspirate with branching pseudopapillary fragments in a straight line, L shape, and C shape; central delicate capillaries (black arrows) surrounded by discohesive small- to medium-sized monomorphic neoplastic cells (Case #7, EUS-FNA, Diff-Quik, original magnification ×100). (**B**) Delicate central capillary network (black arrow) and discohesive cercariform cells (red arrows) (Case #3, Papanicolaou, EUS-FNA, original magnification ×200). (**C**) Relatively uniform plasmacytoid single neoplastic cells, with some showing a cytoplasmic tail (red arrows) (“cercariform cell”) (Case #3, EUS-FNA, Diff-Quik, original magnification ×200). (**D**) Monomorphic neoplastic cells with extracellular metachromatic magenta material (black arrows). Note the foamy macrophage in the lower field (Case #3, EUS-FNA, Diff-Quik, original magnification ×400). (**E**) An extreme example of prominent extracellular metachromatic magenta material (black arrows), corresponding to the myxoid stroma seen in (**F**) (Case #6, EUS-FNA, Diff-Quik, original magnification ×200). (**F**) Solid-pseudopapillary neoplasm with prominent myxoid fibrovascular stroma (black arrow) and paranuclear vacuoles (red arrow) (Case #6, resection, H&E, original magnification ×400). (**G**) Neoplastic cells with intracytoplasmic vacuoles (black arrows) on ROSE, interpreted as “atypical cells with mucinous features”, corresponding to the intracytoplasmic eosinophilic hyaline globules seen on the H&E stain in (**H**) (Case #1, EUS-FNA, Diff-Quik, original magnification ×400). (**H**) Neoplastic cells with intracytoplasmic eosinophilic hyaline globules (black arrows) (Case #1, EUS-FNA, cell block, H&E, original magnification ×200).

**Figure 2:**
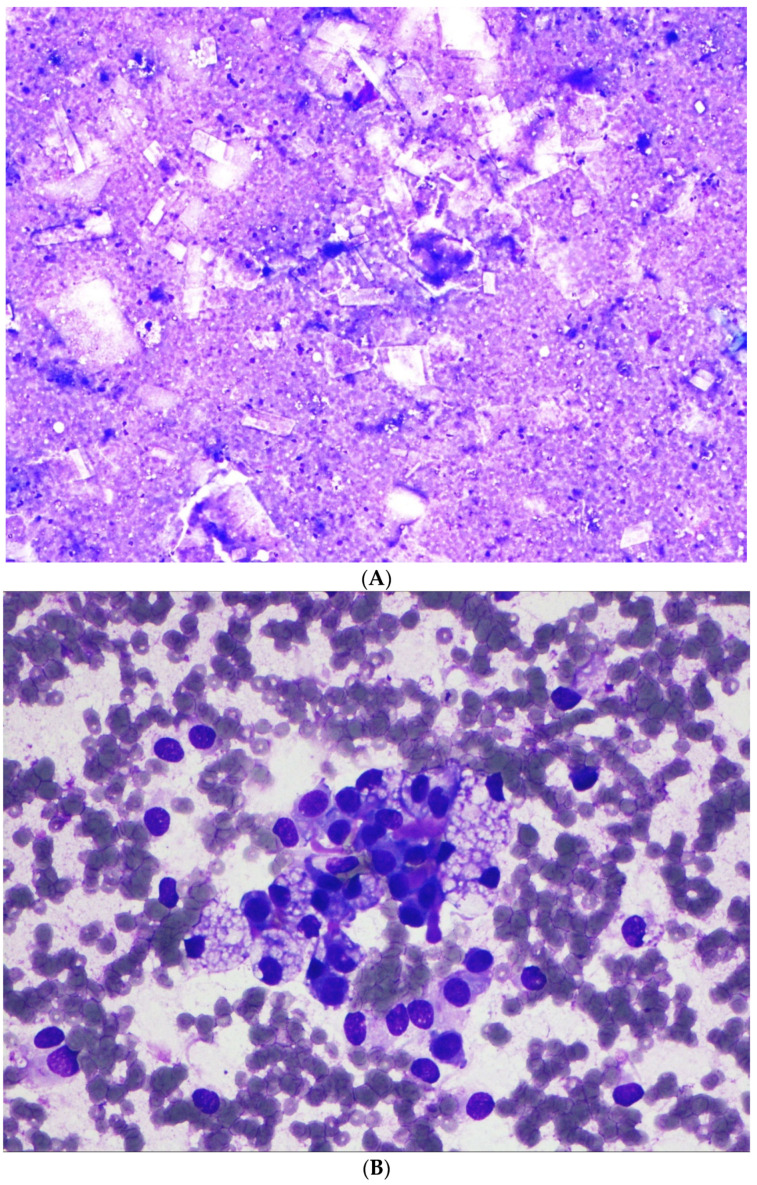

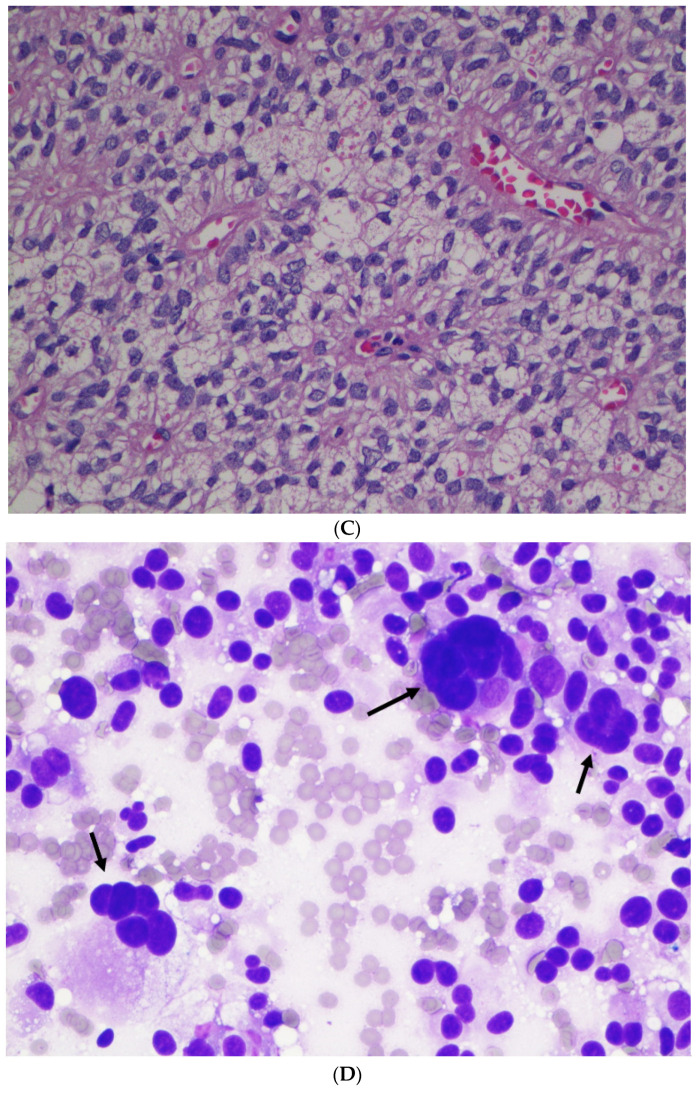

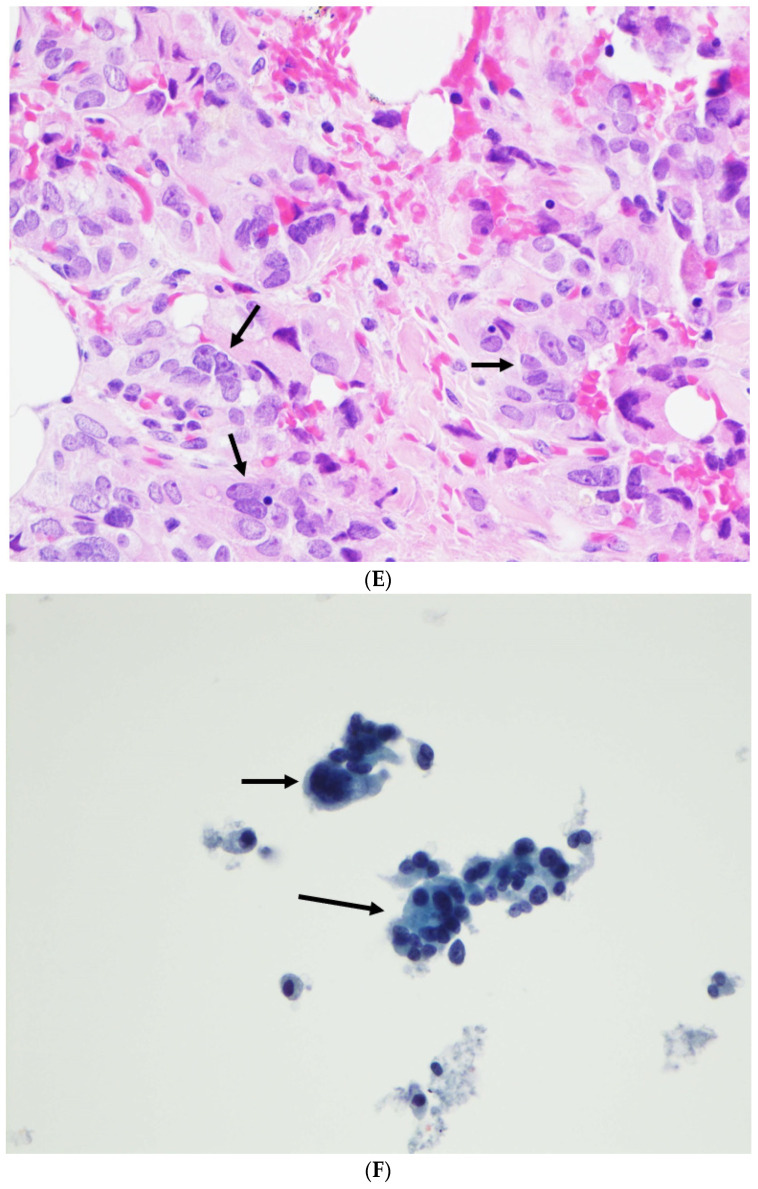

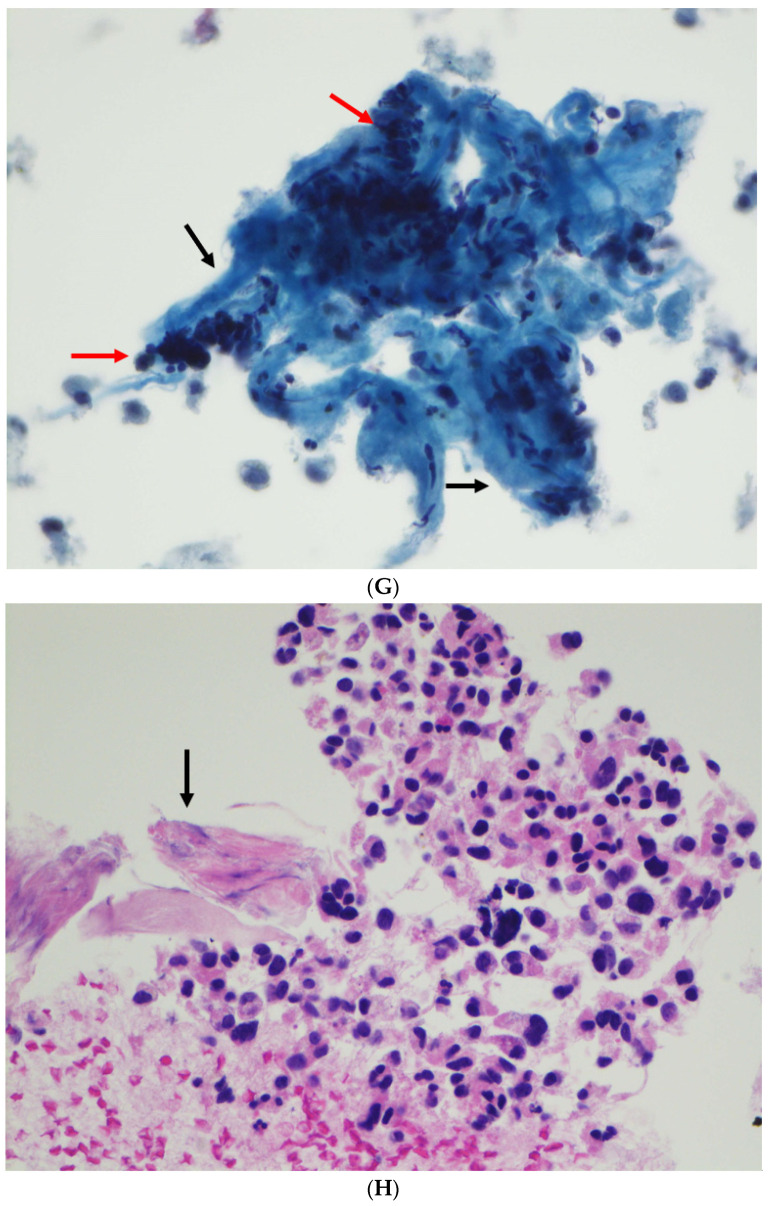
(**A**) Cholesterol crystals (Case #7, EUS-FNA, Diff-Quik, original magnification ×100). (**B**) Prominent clear cell changes (Case #9, EUS-FNA, Diff-Quik, original magnification ×400). (**C**) Prominent clear cell changes (Case #9, EUS-FNA, cell block, H&E, original magnification ×400). (**D**) Monomorphic neoplastic cells in contrast to large pleomorphic atypical multinucleated giant cells (black arrows) with a vacuolated cytoplasm (Case #12, EUS-FNA, Diff-Quik, original magnification ×400). (**E**) Pleomorphic atypical multinucleated giant cells (black arrows) with nuclear irregularity (Case #12, EUS-FNA, cell block, H&E, original magnification ×400). (**F**) Separate case showing atypical multinucleated giant cells (black arrows) (Case #14, EUS-FNA, Papanicolaou, original magnification ×200). (**G**) Hyalinized acellular stroma (black arrows) associated with atypical multinucleated giant cells (red arrows) (Case #14, EUS-FNA, Papanicolaou, original magnification ×200). (**H**) Hyperchromatic atypical multinucleated giant cells and acellular hyaline and myxoid fibrovascular stroma (black arrow) (Case #14, EUS-FNA, cell block, H&E, original magnification ×200).

Of the 22 cases, 5 only had surgical pathology material without a prior cytological diagnosis and were therefore excluded from further analysis. All of the remaining 17 cases were confirmed to be SPN on resection. Based on our institution’s practice, all cases had Diff-Quik-stained smears. Papanicolaou-stained direct smears or ThinPrep material were available for review in two cases. A detailed review of the cytological features was undertaken (summarized in Table 2). All but one showed high cellularity. All 17 cases showed branching papillary-like cellular clusters (Figure 1A,B). All but one case showed central capillaries within the papillary clusters (Figure 1A,B). Similarly, all but one case showed myxoid fibrovascular stromal fragments (Figure 1D–F), and all but one showed numerous loosely cohesive or single monomorphic neoplastic cells (Figure 1C). These monomorphic cells had a plasmacytoid morphology. Tumor cells showed fine nuclear chromatin and nuclear grooves (better seen on Papanicolaou and H&E stains) (Figure 1B,F,H and Figure 2C,E). Binucleation was noted in all cases. Cercariform cells were seen on all smears with high cellularity, but not on touch imprint material (Figure 1B and Figure 2F). Neither necrosis nor mitosis was seen in any of the cases. The above features were therefore considered to be usual or typical features since they were seen in almost all cases.

A few cases showed unusual or atypical features. Background foamy macrophages (Figure 1C,D) and/or cholesterol crystals (Figure 2A) were seen in 10 cases and 1 case, re-spectively. These were considered to be degenerative changes that often accompany cell discohesion and breakdown in pseudopapillary tumors. One case showed a prominent clear cell change (Figure 2B,C), raising a differential diagnosis of metastatic renal cell car-cinoma, or PanNET with clear cell changes. Large atypical multinucleated giant cells were seen in two cases with cytological material (Figure 2D–H). Giant cell nuclei varied from being vesicular with prominent nucleoli to hyperchromatic (Figure 2E,H).

Rapid on-site evaluation (ROSE) was performed in ten cases. This included the eval-uation of smears at the time of EUS-FNA in seven cases, and touch imprints at the time of CT-guided core biopsy in three cases. Lesional material was confirmed at the time of the procedure in all ten cases (Table 1). However, a definite diagnosis of SPN was rendered in only 1 of these 10 cases at ROSE. The differential diagnoses at ROSE included PanNETs and round cell tumors (two cases each). In the latter scenario, material was sent for flow cytometry; cells were positive for CD56 and CD10, and negative for CD45. One case was felt to have atypical cells with mucinous features. In reviewing the slides created at ROSE, we found that cercariform cells were seen in smears but not in touch imprints, leading us to hypothesize that this peculiar morphology may be the result of a stretching artefact caused by mechanical “strain” on the cell at the time of smearing.

Immunohistochemical stains were performed in 16 of the 17 cases with cytological material (Table 3), either for the confirmation of diagnosis or to resolve differential diag-noses. Where immunohistochemical stains were performed, 100% of cases were positive for nuclear β-catenin, CD10, α1-antitrypsin, CD56, cyclin-D1, progesterone receptor (PR), and vimentin. Interestingly, 46% of cases were positive for synaptophysin, but none were positive for chromogranin. Only 1 of the 11 cases was positive for pancytokeratin (CK AE1/AE3). Tumors showed a low Ki-67 proliferation index (not greater than 5%). Im-munostaining for CD99 was only performed on Case #15 and was negative.

The available clinical follow-ups ranged from 2 to 177 months, with a median of 69 months. Nineteen patients were alive and tested negative for recurrent or metastatic SPN. Three patients had no available clinical follow-up information.

## 4. Discussions

Pancreatic SPNs are rare, accounting for less than 3% of pancreatic exocrine neoplasms [1,2,3,4,5,6]. We found only 22 cases in our database in a large tertiary care community practice over a 15-year period (to provide perspective, we have over 150 cases of EUS-FNA of pancreatic masses every year). These tumors are generally thought to affect young females. Analysis of the SEER database revealed that SPNs had a bimodal age–frequency distribution in females (early onset peak at 28 y and late onset peak at 62 y), whereas male patients had a unimodal peak (64 years) [16]. While we did find a wide age range among our female patients, unlike the SEER database, all our male patients were young.

The symptoms of most patients with SPN are reported to be nonspecific, and some SPNs are incidentally discovered [1,2,17]. Most of our patients presented with abdominal pain or discomfort, and only in one case was the tumor incidentally discovered. The tumors in our cohort had a median dimension of 4.0 cm (mean: 4.7 cm), which is smaller than the 6.6 to 8 cm size in other studies [4,6,8,15], and comparable to a recent study [18]. Tumors are usually solitary and more frequently reported in the tail [1,2,4], similar to our cases.

Cytology has come to be a mainstay in the work-up and diagnosis of pancreatic tumors [7,8,9,10,11]. The cytological identification of SPN is important in order to prevent unnecessary aggressive treatments, including preoperative chemoradiation and radical surgery. With a sufficient sample, the diagnosis of SPN is usually straightforward since it has characteristic cytological features and IHC profiles [7,8,9,10,11,12,13,14,15,18]. The classical cytological features of SPN include pseudopapillary clusters with central fibrovascular cores surrounded by monomorphic neoplastic cells, with a small or moderate amount of cytoplasm [7,8,9,10,11,12,13,14,15]. In this study, pseudopapillary clusters with thin central capillaries were seen in all but one case. A varied amount of background extracellular myxoid hyalinized fibrovascular material was identified in all cases. Neoplastic cells had a mostly monomorphic plasmacytoid morphology with fine nuclear chromatin, without prominent nucleoli. The nuclear grooves or folds were better seen with Papanicolaou or H&E stains [14].

The major differential diagnosis we encountered was pancreatic neuroendocrine tumor (PanNET). PanNET was mentioned as a preliminary diagnosis in a few cases at ROSE. In fact, Case #3 was diagnosed as PanNET in the original final FNA report. The prominent pseudopapillary clusters with central capillaries, myxoid fibrovascular core/extracellular material, and cercariform cells offered clues for SPN. In contrast, PanNET neoplastic cells show salt and pepper nuclear chromatin, arranged in a single form or rosettes [4,8,14].

Case #1 showed abundant intracytoplasmic vacuoles on Diff-Quik-stained direct smears, leading to a ROSE diagnosis of “atypical cells with mucinous features.” Given that this was a 65-year-old patient, the main consideration was of an adenocarcinoma. In our opinion, these intracytoplasmic vacuoles are intracellular eosinophilic hyaline globules that are immunoreactive for α1-antitrypsin. These findings are consistent with other studies [10,11]. One case showed clear cells resembling clear cell renal cell carcinoma [19,20], and the differential diagnosis was resolved by immunohistochemistry for PAX8: neoplastic cells were positive for β-catenin and negative for PAX-8.

As seen in our study, SPNs are typically immunoreactive for nuclear β-catenin, PR, cyclin-D1, CD10, α1-antitrypsin, and vimentin. The expression of pancytokeratin and synaptophysin is variable, while neoplastic cells are negative for chromogranin. Nuclear expression of β-catenin is specific to SPNs [21,22] due to somatic mutations in CTNNB1 exon three hotspots. PanNET, the most likely differential, would be positive for pancytokeratin, synaptophysin, and chromogranin. We propose a limited IHC panel including three nuclear stains (beta-catenin, PR, and cyclin-D1) and three cytoplasmic stains (CK AE1/AE3, synaptophysin, and chromogranin) to exclude NETs.

**Figure 3:**
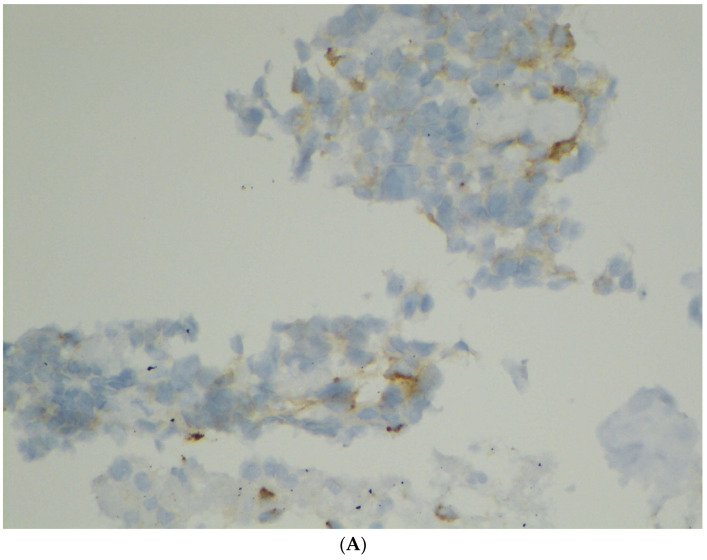

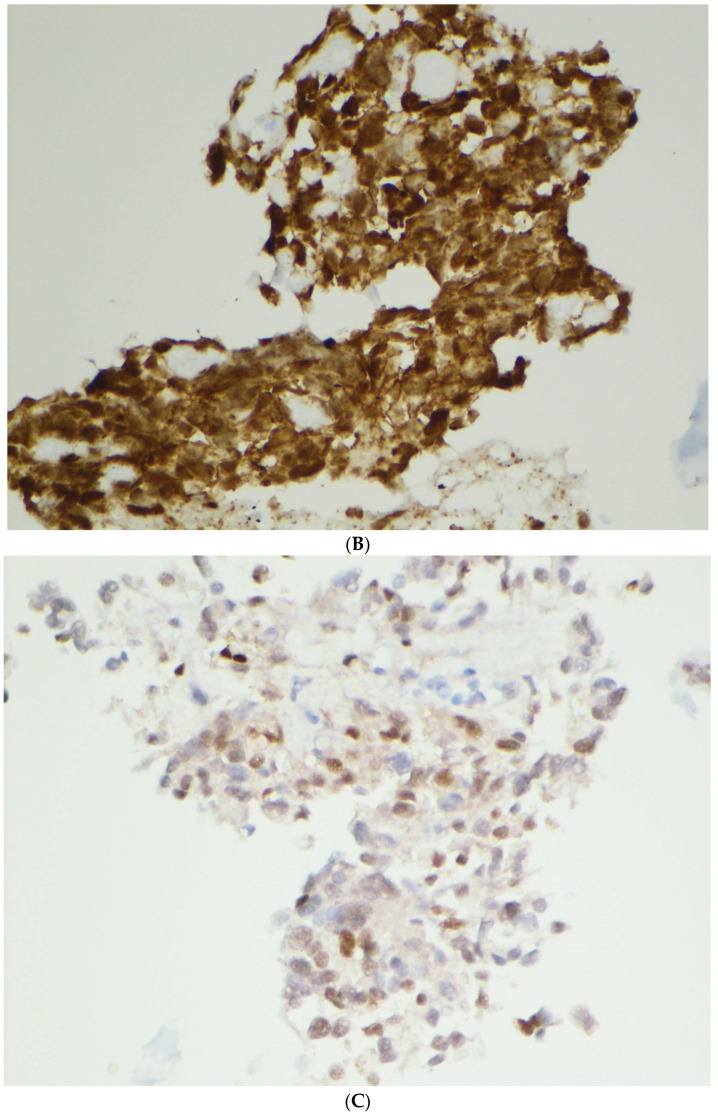

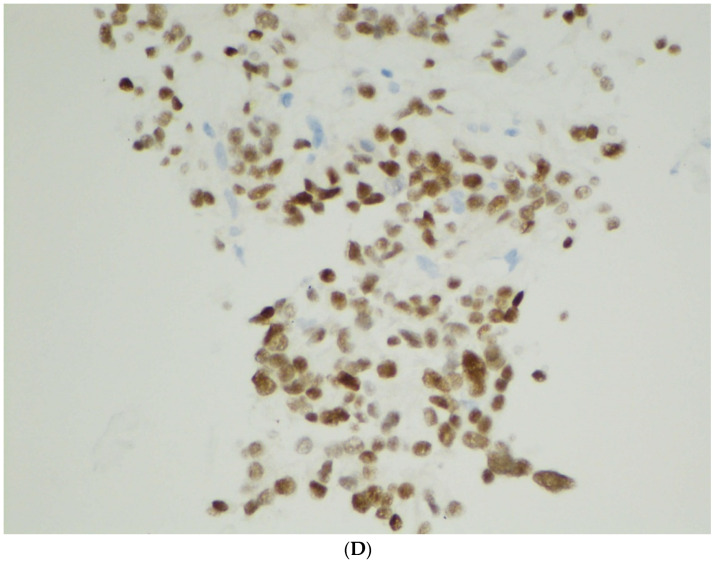
(**A**) Weak immunostaining for synaptophysin (Case #14, cell block, original magnification ×400). (**B**) Strong nuclear and cytoplasmic immunostaining for β-catenin (Case #14, cell block, original magnification ×400). (**C**) Nuclear immunostaining for cyclin-D1 (Case #14, cell block, original magnification ×400). (**D**) Nuclear immunostaining for progesterone receptor (Case #14, cell block, original magnification ×400).

In this study, we identified three cases (Case #12, 14, and 20) with atypical multinucleated giant cells. The findings were alarming, since malignant neoplasms with a worse prognosis entered the differential diagnosis, including PanNET with endocrine atypia, pancreatic carcinoma, and metastatic malignancy. As can be seen in Figure 3, the large atypical multinucleated neoplastic cells were immunoreactive for nuclear β-catenin, cyclin-D1, and PR, while they were negative for CK AE1/AE3 and chromogranin, with a low proliferation index, confirming the diagnosis of SPN. Similar giant cells have recently been described in a few reports [23,24,25]. Interestingly, all of our cases with atypical cells occurred in relatively older patients, leading to the consideration of pancreatic carcinoma. It is therefore important to remember that SPNs can occur in older individuals and can present with degenerative atypia. Unlike pancreatic adenocarcinomas and NETs, SPNs are low-grade tumors with an excellent prognosis, as in our cohort.

## 5. Conclusions

We have presented our experience with cytological diagnoses of SPN in a well-characterized cohort of 22 patients with histological correlation and follow-up data. These tumors occur over a wide range and show varied cytological features. SPNs can be confidently diagnosed even on limited cytologic material. Recognizing associated degenerative changes is crucial in avoiding a misdiagnosis. A strong suspicion irrespective of age and gender, attention to cytological details, and the application of a limited IHC panel can be helpful in establishing a definitive diagnosis, and patient management.

## Figures and Tables

**Table 1:** SPN: clinical presentation, radiological/gross features, and cytological correlation.

Case	Age (Years)	Sex	Site	Radiology/Gross Features	Size (cm)	ROSE Diagnosis	FNA/Biopsy Diagnosis
1	65	F	Body	Solid and cystic	4.0	Atypical cells with mucinous features	SPN
2	39	F	Tail	Cystic	3.0	Serous papillary lesion	SPN
3	28	F	Body	Solid and cystic	4.0	NA	SPN
4	26	F	Tail	Solid and cystic	4.0	NA	SPN
5	33	F	Body	Solid and cystic	6.5	Lesional tissue	SPN
6	26	F	Tail	Solid and cystic	1.5	Suspicious for SPN vs. PanNET	SPN
7	24	F	Tail	Solid and cystic	4.0	SPN	SPN
8	26	M	Tail	Solid and cystic	3.5	NA	SPN
9	25	M	Body	Solid	2.2	Favor PanNET	SPN
10	29	F	Body	Solid	2.9	Lesional tissue	SPN
11	23	M	Body	Solid and cystic	4.8	NA	SPN
12	41	F	Tail	Solid and cystic	2.2	NA	SPN
13	20	F	Head	Solid	6.0	NA	SPN
14	73	F	Body	Solid and cystic	1.2	NA	SPN
15	21	F	Tail	Solid and cystic	15	Round cells	SPN (biopsy)
16	21	F	Tail	Solid and cystic	12	Lesional cells	SPN (biopsy)
17	47	F	Body	Solid and cystic	4.7	Tumor	SPN (biopsy)
18	46	F	Tail	Solid and cystic	4.5	NA	NA
19	14	F	Tail	Solid and cystic	3.7	NA	NA
20	71	F	Body	Solid and cystic	15	NA	NA
21	12	F	Tail	Solid and cystic	3.5	NA	NA
22	16	F	Tail	Solid and cystic	3.5	NA	NA

Abbreviations: ROSE, rapid on-site evaluation; NA, not available; SPN, solid-pseudopapillary neoplasm; PanNET, pancreatic neuroendocrine tumor; FNA, fine needle aspiration.

**Table 2:** Cytomorphology of 17 SPNs of the pancreas on FNA smears/touch prints and cell block/biopsy.

Case	Papillation	Monomorphic Cells	Central Capillaries	Nuclear Groove	Myxoid Fibrovascular Stroma	Cytoplasmic Hyaline Globules	Atypical Multinucleated Cells
1	+	+	+	+	+	+	-
2	+	+	+	+	+	+	-
3	+	+	+	+	+	+	-
4	+	+	+	+	+	+	-
5	+	+	+	+	+	+	-
6	+	+	+	+	+	+	-
7	+	+	+	+	+	+	-
8	+	+	+	+	-	+	-
9	+	+	+	+	+	+	-
10	+	+	+	+	+	+	-
11	+	+	+	+	+	+	-
12	+	+	+	+	+	+	+
13	+	+	+	+	+	+	-
14	+	-	-	+	+	+	+
15	+	+	+	+	+	+	-
16	+	+	+	+	+	+	-
17	+	+	+	+	+	+	-

**Table 3:** SPN: immunohistochemistry profile in cases with cytological material.

Case	β-Cat	CyD1	PR	CD56	SYN	CHR	CD10	CK	VIM	AAT	Ki-67
1	+	NA	NA	+	Focal	NA	+	-	+	+	NA
2	+	NA	NA	+	NA	NA	+	NA	+	+	NA
3	NA	NA	NA	+	-	-	+	NA	NA	NA	NA
4	+	NA	NA	+	Focal	-	+	-	+	+	NA
5	+	NA	NA	+	Focal	-	+	-	+	+	NA
6	+	NA	NA	NA	-	-	NA	NA	NA	+	NA
7	NA	NA	NA	NA	NA	NA	NA	NA	NA	+	NA
8	+	NA	NA	+	NA	NA	+	NA	NA	+	NA
9	+	NA	NA	NA	Focal	-	+	+	NA	+	<5%
10	+	NA	+	+	-	-	+	-	NA	NA	<1%
11	+	+	+	NA	-	-	+	-	NA	NA	<5%
12	+	NA	NA	NA	-	-	NA	-	NA	NA	<5%
13	+	+	+	NA	Focal	-	NA	-	NA	NA	5%
14	+	+	+	NA	Focal	-	NA	-	NA	NA	1%
15	+	+	+	+	-	-	+	-	NA	+	NA
16	+	+	+	NA	-	-	+	NA	+	NA	NA
17	+	+	NA	NA	NA	NA	+	-	NA	NA	NA
Total	15/15 100%	6/6 100%	6/6 100%	8/8 100%	6/13 46%	0/12 0%	12/12 100%	1/11 9%	5/5 100%	9/9 100%	

Abbreviations: β-Cat, β-catenin; CyD1, cyclin-D1; PR, progesterone receptor; SYN, synaptophysin; CHR, chromogranin; CK, cytokeratin AE1/AE3; VIM, vimentin; AAT, α1-antitrypsin; NA, not available.

## Data Availability

This study did not report any data.
